# Progress in State Medicine

**Published:** 1902-01-04

**Authors:** 


### PROGRESS IN STATE MEDICINE.

**Tuberculosis.**—The Report[1] presented to the second general meeting of the members of the National Association for the Prevention of Consumption showed steady progress. New branches had been formed in Liverpool and Manchester, and others were in course of formation. A pamphlet had been issued showing that 21 towns and districts have adopted voluntary notification of consumption, and about the same number of Boards of Guardians and Town Councils have made provision for open-air treatment of their poorer inhabitants. A further list of 58 sanatoria in full working order, with all particulars had also been published for the information of medical practitioners. The total cost of treating 36 patients at the Westmorland Sanatorium amounted to £1,264 5s. 10d. The Report also contains an account of the satisfactory result of the working during three months of the plan recently adopted by the three Liverpool Boards of Guardians of making it a condition for granting relief that the family should move to more wholesome quarters, where the consumptive patient could have a separate room to sleep in, and could also adopt simple but efficient precautions for the prevention of infection through the sputum. The increase in the grant for relief necessary to cover a separate sleeping room was found to average only about 1s. 3d. a week. The right of guardians to do this seemed to be fully covered by their legal duty of giving "adequate relief."

At the Tuberculosis Conference in Canada, Stewart[2] pointed out that the greatest instrument to be relied on for educating the people was the public press. The combined efforts of the press for a short time would do much more than the individual efforts of decades. Not newspapers which on one page contained an article pointing out the true nature and treatment of tuberculosis while another was given over to the advertisements of quacks pretending to cure the disease, but a pure press. The most important measure was the establishment of sanatoria and homes for the poor: sanatoria for cases of incipient disease, and homes for advanced cases where cure was improbable. This was a duty of the State, as also of enacting laws to prevent those affected with tuberculosis from working at trades in which there was a danger of contamination of food and clothing. Adami,[3] referring to the powers already possessed by Government of inspecting and condemning imported animals suffering from tuberculosis, declared that new settlers showing signs of the disease should be prevented from entering the country. Experimental sanatoria should be established in each of the several Canadian climes, in which the therapeutical effects of such climes could be scientifically studied. Arrangements should be made with the railway companies to segregate persons obviously consumptive from the rest of the travelling public. Government should make an experiment in a well-defined area to demonstrate that tuberculosis could be wholly eradicated from herds.

Cameron[4] draws attention to the fact that whilst in England many towns have voluntary notification of consumption, funds being available for that purpose, in Dublin there is no fund for the notification of any disease save those in the Schedule of the Infectious Diseases Notification Act. The objections made against compulsory notification are chiefly: (1) that it would subject consumptives to practically an isolated life; (2) that it is often not possible to certify positively that a patient is suffering from undoubted phthisis. Points in its favour are that it would enable the sanitary authorities to disinfect rooms which had been vacated by consumptives; useful information in the form of leaflets could be given to their families; disinfectants for the treatment of sputa could be supplied free of charge to the poorer patients. With regard to milk being a vehicle for the propagation of tuberculosis he mentions that both Fisher and Webber doubt that sterilisation as now performed really destroys disease germs, and assert that it decreases the digestibility by alteration produced in its albuminoids and lactose. Experiments should be instituted to settle this point. England is behind other countries in preventive measures. Notification of tuberculosis is now compulsory in Norway as well as in New York. In the latter there has been a reduction of 35 per cent. in the mortality ascribed to tuberculous disease since 1883, the year the notification of these diseases came into force. In Rhode Island the Board of Health are asking £40,000 from the Government for a hospital for consumptives. There is a Bill before the Legislature of New Jersey to provide funds for a similar object. Burdon Sanderson[5] insists that the kind of help to be given to the consumptive bread-earner must be determined by the condition of the individual that we desire to benefit. He should be enabled to make the best of the strength that remains to him. This necessitates firstly that havens of refuge shall be always open to him during his temporary illnesses, and secondly, when the progress of the disease makes work impossible, means should be provided for his sustenance. Every industrial centre should have its hospital or ward solely for progressive cases, such cases to be always liable to dismissal at the discretion of the medical authority in charge. By this means many a patient would be enabled to regain his footing and return to work, if not with renewed vigour with renewed hope and courage. In connection with the more advanced chronic cases the aim should be not so much the relief of suffering as the maintenance of earning power. This can only be accomplished by immunity from labour, accompanied by good air and food. Sanatoria for the poor are a necessity. These are provided in Germany through the compulsory insurance against sickness and old age. In England poverty is the most influential factor accounting for the high phthisical mortality amongst workmen in the prime of life. Comparing persons who earn £1 a week with those who earn £2 to £3 a week, the annual mortality from phthisis is four per 1,000 of the former class as against two per 1,000 of the latter. It is estimated that the life of a consumptive, in easy circumstances, is about seven and a half years from the commencement of the disease, whereas of every 100 workmen in the initial stage of phthisis, earning a little more or less than £1 a week, 50 will have died in three years. Had these 100 persons been treated sanatorially, statistics tend to show that in about 70 per cent. health would have been so far restored that they would have been able to support themselves and their families, and that if their condition had been investigated two years later there would have been some 50 persons still capable of earning their living. Owing to immense expense it is not advisable to copy the German method of State insurance. Sanatoria ought to be self-supporting; maintained by those who benefit by them. The employers of labour should consent to levy upon themselves a contribution proportional to, and deductible, in whole or in part, from the wages paid to their workmen. This unfortunately would exclude the toilers of the poorer class.

Coates[6] experimented to ascertain if infective material in dangerous amount accumulated in houses occupied by consumptive patients. Samples of dust from various situations in the room under investigation were collected with aseptic precautions, care being taken that direct infection with sputum was impossible. This dust was then mixed with sterilised water and used to inoculate guinea-pigs. In many instances the inoculated animals died within 48 hours from septic diseases due to germs other than tubercle bacilli. Those surviving were killed at the end of a month, and a post-mortem, as well as a microscopic examination made of each. The houses examined he divides into two groups, (1) those which were dirty and contained a phthisical patient who spat about the floor; (2) clean houses where the patient behaved in the same manner. Fourteen houses out of 21 in the first group, gave direct experimental proof of the presence of infective dust, which if inhaled could give rise to consumption. In the second group ten houses were examined and infective dust was found in five, showing that ordinary cleanliness is not sufficient to prevent infection of the atmosphere. Further check experiments were made in houses in which it was certain no case of tuberculosis had occurred for three years or more, and which corresponded exactly in condition as to cleanliness with those in the first group. In no case was infective dust found in these houses, thus demonstrating the need for disinfection of houses vacated by consumptives. To test the efficacy of the disinfection as practised in Manchester, namely with chlorinated lime solution, pieces of wall-paper, infected with tuberculous sputum, were pinned to the walls of a room about to be disinfected. These papers, after the process was over, were allowed to dry, and then used to inoculate guinea-pigs. Control papers were used to inoculate other guinea-pigs. In every instance the control papers produced tuberculosis, with negative results with the disinfected papers. Attention[7] is called to the fact that in the poorer class of houses it is the habit of paperhangers to tear away the old paper which is hanging loosely on the wall, and which they can remove without soaking with water. A large amount of dust is thus distributed and inhaled, dust perhaps containing innumerable bacilli from the sputa of a careless consumptive patient. Weatherly[8] is of opinion that the great prevalence and mortality from phthisis in lunatic asylums is due to overcrowding, lack of sufficient pure air and exercise, and a certain quality of dietary. The present tendency of piling building on to building is to be deprecated. Greater facilities are wanted with regard to window ventilation. If a little more money was spent in the quality of the diet and a little less on palatial buildings much would be done to check this disease. Spittoons fastened by locked rings should be fixed in all corridors, wards, and bedrooms. For diagnostic purposes the tuberculin test was important. The sanatoria or blocks for phthisical insane should be built on sandy or gravelly soil, with a south aspect. To build small detached cottages for such a purpose would be a waste of money, whereas a sanatorium of moderate size could be built at £200 a bed, and in such each patient could be given a separate room to sleep in.

[1] Brit. Med. Jour., March 30, 1901. [2] Brit. Med. Jour., April 20, 1901. [3] Ibid. [4] Dublin Med. Soc. Jour., May, 1901. [5] Brit. Med. Jour., July 6, 1901. [6] Sanit. Rec., Nov. 21, 1901. [7] Sanit. Rec., Nov. 21, 1901. [8] Brit. Med. Jour., March 2, 1901.

